# The Integration of the Glutamatergic and the White Matter Hypotheses of Schizophrenia’s Etiology

**DOI:** 10.2174/157015912799362742

**Published:** 2012-03

**Authors:** Alvaro Machado Dias

**Affiliations:** 1 Federal University of São Paulo; 2 Dept. of Neuroimaging in Psychiatry 'LIM-21', University of São Paulo Medical School, Brazil

**Keywords:** Schizophrenia, molecular psychiatry, connectivity, glutamate, white matter.

## Abstract

**Background:**

schizophrenia's endophenotipic profile is not only generally complex, but often varies from case to case. The perspective of trying to define specific anatomic correlates of the syndrome has led to disappointing results. In that context, neurophysiologic hypotheses (e.g. glutamatergic hypothesis) and connectivity hypotheses became prominent. Nevertheless, despite their commitment to the principle of denying 'localist' views and approaching the syndrome's endophenotype from a whole brain perspective, efforts to integrate both have not flourished at this moment in time.

**Objectives:**

This paper aims to introduce a new etiological model that integrates the glutamatergic and the WM (WM) hypotheses of schizophrenia’s etiology. This model proposes to serve as a framework in order to relate to patterns of brain abnormalities from the onset of the syndrome to stages of advanced chronification.****

**Highlights:**

Neurotransmitter abnormalities forego noticeable WM abnormalities. The former, chiefly represented by NMDAR hypo-function and associated molecular cascades, is related to the first signs of cell loss. This process is both directly and indirectly integrated to the underpinning of WM structural abnormalities; not only is the excess of glutamate toxic to the WM, but its disruption is associated to the expression of known genetic risk factors (e.g., NRG-1). A second level of the model develops the idea that abnormal neurotransmission within specific neural populations ('motifs') impair particular cognitive abilities, while subsequent WM structural abnormalities impair the integration of brain functions and multimodality. As a result of this two-stage dynamic, the affected individual progresses from experiencing specific cognitive and psychological deficits, to a condition of cognitive and existential fragmentation, linked to hardly reversible decreases in psychosocial functioning.

## INTRODUCTION

When Kraepelin [1] suggested a role for brain deterioration in the etiology and the progression of dementia praecox, he probably did not have in mind the extent of the debate that was about to start around such an issue. More than a century later, one may note that the general agreement around the existence of biological markers of brain deterioration from schizophrenia's first episode, to its chronic development, does not represent an endpoint to this discussion, as the specificity of such brain impairments remains surrounded by doubts.

Intricacy in this field emerged as a rule, not an exception, and one thing that has proved to be true is that variation among different cohorts can lead to divergent findings. For example, while one meta-analysis, based on general studies of gray matter abnormalities, suggested that the manifestation of schizophrenia is accompanied by gray matter losses in the bilateral temporal medial areas [2], another one indicates that this alteration should not be assumed to be relevant, since it was allegedly reported as 'not relevant' by most experimental studies [3]; this divergence in conclusion probably relates to the fact that the latter study was solely based on first-episode studies, contrary to the other one, which had a less restrictive experimental design. A recent meta-analysis confirmed this dynamic (and progressive) tendency within experimental frameworks [4].

Likewise, in the field of functional studies, things are not so different and suggest caution when reading through papers that present discrepant conclusions about schizophrenia's neural dysfunctions. For example, in relation to cerebral blood flow within the prefrontal cortex during task execution, many studies related schizophrenia to hypofrontal activation in rest and during task execution [5-9], while others found hyperfrontal activation in task execution only [10-14]. As stated by some [13], hyperfrontality is related to positive symptoms, especially ‘ego pathology’, while others [10] relate it to negative symptoms, particularly during task execution. This matches with the perspective that progression (which enhances negativism) might be the key for the divergence.

Up to a certain extent, this conundrum has to do with divergent inclusion criteria, and from that point on, it probably relates with the general structure of the methodologies that are applied to select psychiatric cohorts in general; subjects invited to participate in most psychiatric studies are selected by means of the application of diagnostic instruments that hold the assumption that nosographic units can be discriminated by the process of clustering common symptoms (in other words, they are 'descriptive', as it applies to DSM; [15]), while it might be the case that, among cases selected this way, endophenotipic divergence is more of a rule than an exception.

In the same vein, chronification might be different from one case to the other, despite the outward showing of a pattern provided by the process of clustering cases in terms of descriptive diagnoses and, in a broader sense, considering schizophrenic manifestations in terms of their positive, negative, and cognitive dimensions.

We hypothesize that endophenotipic differences among subjects diagnosed with schizophrenia also have a role in establishing the contention that has been established in relation to the precise number of dimensions by which the syndrome must be approached - and the picture that we find today is that some authors argue that schizophrenia should be clustered by a two-dimensional diagnosis [16], a three-dimensional diagnosis [17-20] and a five-dimensional diagnosis [21, 22].

As this picture suggests, there is an acute difficulty in defining a model capable of integrating different patterns of brain abnormalities, from the onset, to more chronic stages of the syndrome. It is feasible to assume that this task is one of the main challenges in schizophrenia research and that the establishment of such an empirically-based model of endophenotipic deterioration would aid not only the understanding of the syndrome's underlying structure, but also solve matters related to its nosological and nosographic classification.

Currently, any attempt to define models as the ones that may be included in the above perspective posits a methodological issue: in opposition to many canonical conceptions of the biological basis of schizophrenia, it is becoming clear that it is not feasible to grasp a clear picture of what is dynamically happening with the brain. When the focus is restricted to information regarding dysfunctions in 'localist' brain networks, that is, if the leitmotiv is reduced to the analysis of patterns of gray matter abnormalities in regions of interest, and as defined in light of the discrimination of neural networks/areas as they appear in brain atlases. It is becoming clear that a ‘transmission paradigm’ cannot be excluded from such global models of endophenotipic dysfunction - as Friston and Frith [23] proposed with their hypothesis of schizophrenia as a ‘disconnection syndrome’.

However, things are not so simple when considering the progression from onset to chronification, due to the fact that white matter (WM) abnormalities are not usually found at the onset of schizophrenic manifestations (usually in early adulthood). As we see it, and we will try to endorse this as we go along with this paper, anatomical disconnection is an important piece in schizophrenia’s endophenotipic puzzle, but not a necessary and sufficient variable to explain the most basic event: the onset of the syndrome, which precisely must be integrated to chronification (in terms of endophenotipic abnormalities) in a more complex model.

Remarkably, there is another type of 'connectivity' -from cell to cell- which is indeed impaired from the onset of the syndrome, and which must be integrated to WM abnormalities in a more omnibus model: schizophrenia is chiefly related to neurochemical abnormalities that affect cellular signaling at the synaptic level, and that are associated with some of the most robust genetic markers of hereditability, like Neurogulin-1 (NRG1). As most readers of this Journal are probably aware, the domain of the field of molecular psychiatry that deals with the psychoses' neurochemical bases has endorsed the relatively new schizophrenia's 'glutamatergic hypothesis' with enthusiasm, in opposition to the classic dopaminergic hypothesis, which is losing field in pace with the expansion of research. 

In mainstream psychiatric research, these two types of 'transmission paradigms' are usually treated as separated topics, despite their convergence around the idea of putting forward alternatives to older studies that have tried to reduce the emergence and chronification of schizophrenia to the presence of a predefined set of regional brain dysfunctions ('localist' studies). From our standpoint, this assumption of independence has to do with the difficulty of integrating both types of disconnection in terms of 1. a common leitmotiv; 2. a dialectic relation, by which an integrative view is expected to emerge. This paper aims to introduce a new etiological hypothesis, with the potential to fulfill this gap.

This hypothesis is based on the premise that a neuroanatomic and a neurophysiologic approach to schizophrenia's endophenotype can and should be associated with the establishment of a more complete and dynamic etiological hypothesis, and that these should be respectively represented by the assumption that the syndrome relates to connectivity problems (WM dysfunctions) and the premise that there is an excess of glutamate in the brain of the affected subjects.

### 1 WM Studies: Recent Findings Suggest a Neuron-Glia Network 

The CNS is made of grey matter and WM. The former consists of cell bodies, which take a central role in information processing through the implementation of the ‘all or nothing’ dynamic (rate of firing) of the action potentials; glial cells; and capillaries. The latter consists of axons, glia and microglia; its core function is to transmit the action potentials from one cell to others. Among the glial cells of the CNS, the oligodendrocites are responsible for producing myelin. Axons and oligodendrocites are considered the core elements of WM.

To grasp a profound sense of WM participation in a whole brain information process, it is interesting to approach it in light of the concept of 'motif' (close related neuron populations) and to bear in mind that information spreads both within and between motifs. Not all motifs have the same overall importance: integrative/multimodal cellular populations represent very important motifs [24] (e.g. PFC). In a nut shell, what makes the PFC an important motif is its high degree of connectivity; it is a pathway to several less connected inputs, in relation to which it represents an important pivot - much in the sense that a very popular site represents a necessary pivot to other, smaller sites, which can be drastically affected by the shutdown of the major one. Different brain regions have different (and relatively stable) patterns of local connectivity and inter-motif connectivity, which can be used to rethink the consequences of local abnormalities in terms of a transmission paradigm [25]. WM implements small-world principles by two means: 1. connectivity within motifs; 2. connection of different motifs, either directly or through higher order neuronal assemblies.

In general, the synapses/neuron relation reveals a high degree of overall clustering, which has lead some authors to assume that the brain represents a ‘small-world’ network [24, 26-30], with a low degree of separation between any two CNS neurons and a scale-free organization (in a nutshell: most neurons tend to have a relatively similar importance/number of incoming and out-coming connections, while some have an enormous amount of connections, as it is precisely the case for PFC neurons).

Furthermore, an important aspect for the current model that we are going to propose, is the perspective that this higher order of integrative neuronal populations (the aforementioned 'pivots', which can also be called ‘hubs’) can be divided into two types: the ones that integrate more closely related assemblies (provincial hubs) and the ones that integrate multimodal networks (connector hubs) [24]; damage to provincial hubs decrease small-world indexes, while damage to the connectors increase them in local assemblies [24].

In terms of WM tracts, this means that abnormalities in tracts that integrate different populations of neurons (e.g. frontotemporal pathways) may have as a consequence an increase in the demands over local networks, and the other way around. As we move on to the cognitive level, it is also notable that individual differences in WM structure are usually associated with general neuropsychological traits as fluid intelligence, reactional time [31-33] and executive functioning [34], which express the principle that overall connectivity plays a prominent role as interconnectivity.

In terms of the neurophysiological structure of WM, it was believed until recently that chemical transmission was exclusive to grey matter. This premise was tied to the conception of axons as ‘cables’ - a paradigm also inspired by the fact that the fast release of neurotransmitters requires a very complex specialization within the synaptic cleft, which would only appear at the junction of very complex cells. “A complete set of these proteins is concentrated only in a specialized area of the presynaptic membrane that is directly opposed to the neurotransmitter receptor apparatus of the postsynaptic density” [35], (p.311).

Currently, this paradigm is changing, giving rise to the perspective of a neuron-glia network [36]. First, it was demonstrated that there is an existence of synapses between pyramidal and oligodendrocyte precursor cells of the hippocampus (mature and immature cells) [37]. Further studies with rats revealed that glutamate is released along axons in the WM [35]. Moreover, it was confirmed that oligodendrocytes from the cerebellum and corpus callosum WM could be divided into two types, regular ones, and those that could fire action potentials [38]. Contrary to older dogmas, it was shown that they express NMDA receptors [39, 40]; antibody labels associated with blockage techniques (Mg^2+ ^and ifenprodil block) suggested that the target receptors might display NR1, NR2C and NR3 subunits [41]. Finally, glutamate receptors have been revealed in the premature WM of fetus [42].

### 2 Functional Studies are Suggesting that WM Abnormalities are Associated with Neuropsychological Deficits in Schizophrenia

In the field of functional studies, WM tracts are studied with a special magnetic resonance technique named diffusion tensor imaging (DTI), which takes advantage of the fact that the tracts are oriented by diffusion direction, and thus that (among other features) they can be analyzed in terms of the orientation and quantification of the water dispersion (anisotropy). Diffusion rates are positively correlated to the myelination of the tract; in that sense, DTI is to WM what MRI currently represents to functional studies of grey matter.

Another technique that can be associated with it is the magnetization transfer ratio (MTR), which selectively measures the state of some macromolecules and is especially sensible to myelin protein alterations; the association of MTR and DTI has amplified the detection of connectivity abnormalities [43, 44]. A DTI study with normal subjects concluded that WM in the right posterior inferior longitudinal fasciculus is associated with cognitive capacity (defined by intelligence tests, years of study and reading abilities) among adults [45], children [46] and the elderly [47]. Another study concluded that with quantities of metabolite N-acetylaspartate on the left occipito-parietal, WM predicted intellectual performance [48], suggesting a complex multimodal structure of general intelligence, associated with WM transmission; many studies suggested that myelination decreases with aging, and that this might be related to a decrease in cognitive capacities.

Full blown myelination is a process that occurs in late adolescence. Examining the Yakovlev Collection of normal brains, Benes [49] showed that associative (multimodal) cortical areas gain robust myelination only by the second decade of life, precisely the period of a higher risk in the development of schizophrenia. The first WM study of the brain in schizophrenic patients was delivered in 1998 [50]. The authors used diffusion anisotropy, associated with PETscan, to analyze fronto-striatal connectivity and found that it was impaired in relation to controls. More recently, studies have confirmed parietal and fronto-parietal alterations [51-53], temporal, hippocampal and fronto-temporal [53-60], occipital and fronto-occipital [43, 57, 59], frontal and generally wide-spread alterations [43, 61, 62]. In accordance with those findings, it was revealed that small-world indexes are reduced in schizophrenia [63-67], particularly within frontal, temporal and parietal cortex; although a recent study, that applied graph theoretical tools associated with the MRI data of 203 patients and 259 healthy controls, suggested that this difference might be moderated [68]. 

It is not easy to define the neuropsychological significance of the findings, and thus studies diverge about their core features. One thing that is important to have in mind is that reduced fractional anisotropy can have at least two meanings. A reduced number of axons and reduced myelination, and that currently, it is not possible to distinguish between the two using DTI [69]. In both senses, decreased connectivity is associated with a plethora of sensorial and cognitive dysfunctions (e.g. specific visual deficits [70]); temporal WM and fronto-temporal WM abnormalities are associated with episodic memory deficits [34, 71, 72]; voice hallucinations (VHs) might also be related to fronto-temporal abnormalities [58] and/or to fronto-parietal and corpus callosum tract abnormalities [73]; temporal and occipital WM abnormalities might be associated with visual hallucinations [59], and more general abnormalities (within fiber originating from thalamus, cingulate gyrus, and cortical association areas), might relate to selective attention and executive functioning [34, 72].

### 3 The Glutamate Hypothesis of Schizophrenia’s Etiology is Becoming Paradigmatic

The canonical neurophysiological paradigm about schizophrenia’s etiology was, to recent times, that the disorder was due to a dopaminergic imbalance. Experimentally, it was supported by findings showing that excesses of sub-cortical dopamine (mainly D2) are associated with positive symptoms, while negative and cognitive symptoms are related to diminished dopamine release in different cortical sites [74, 75] (particularly with the frontal, temporal and parietal lobes). In broader clinical terms, its main support was an understanding of the mechanism or action of antipsychotic medication [76], in so much as drugs like amphetamines can exacerbate psychotic symptoms in schizophrenic patients.

Nevertheless, in much the same sense as to what happened in relation to the consolidation of white-grey matter models of schizophrenia (implicit within Kraepelin’s conceptions), a relative old idea (from the eighties [77]) has gained power and is driving a change in the basic neurochemical paradigms in the direction of a broader conception. Pragmatically supported by the disappointment of the dopaminergic paradigm, as the medications (typical and atypical antipsychotics) do not adequately control negative and cognitive symptoms, thus they are not as effective as it would be supposed in the case that schizophrenia was purely the expression of a dopaminergic imbalance.

This new perspective attributes a prominent role to glutamatergic molecular cascades and especially to* N*-methyl-D-aspartate receptors (NMDAR)^1^. These are a type of ionotropic receptor acutely implicated in the generation of slow post-synaptic excitatory potentials (EPSPs), and thus fundamental to the global process of information processing/transmission related to complex cognition and the acquisition of higher order cognitive abilities.

The foundations of this perspective go back to the findings that phencyclidine (which is a street drug commonly called PCP) mimics the main symptoms of schizophrenia, by interfering in the glutamatergic transmission. Animal models of schizophrenia based on PCP administration (in rats) have lead to the hypothesis that mesocorticolimbic dopaminergic deregulations could be due to NMDAR dysfunctions [78]. 

Additionally, it was discovered that ketamine produces the same effects, by also blocking the NMDAR function. Recent computerized analysis of the speech of normal subjects under the effect of ketamine and that of the speech of individuals with schizophrenia revealed significant similarities [79]. Olney and collaborators proposed that the block of the NMDAR receptors at glutamatergic metabotropic transmission sites led to an excessive release of acetylcholine that could also participate in a toxic cascade over the cortical cells [80].

The NMDA receptors act over negative feedback loops within inter-neurons and thus their hypofunction overstresses the pyramidal cells [81]. Precisely as Holcomb and collaborators had emphasized [82], the receptor’s hypofunction leads to a GABAergic hypofunction (GABA is the major inhibitory neurotransmitter), and thus to a subsequent excessive glutamatergic activity in wide spread brain sites due to the suppression of the inhibitory GABAergic cascades. 

Preclinical attempts to enhance the activity of glutamatergic receptors have produced promising effects (specially targeting the G-protein coupled Glu receptors) [83]. Currently, there is at least one drug created to block NMDA receptors under development which is surrounded by great expectancy (LY2140023, from Lilly Laboratories [84]).

Much in the same way as happens in WM studies, it is not easy to grasp a precise picture of neuropsychological correlates to NMDAR dysfunction. Glutamate is the main excitatory neurotransmitter in the brain, and thus participates in innumerous processes, in terms of which, we can point to innumerous findings in all the domains of the affective/cognitive spectrum. Just to point out a few in terms of sensorial abnormalities, the glutamatergic hypothesis has been linked to specific visual deficits (e.g. reduced contrast), and has also been associated with abnormal magnocellular evoked potentials in the early-stages of visual processing [70]. Another study replicated this finding and suggested that it might also be related to working memory deficits [85]. Verbal learning [86] and sustained attention deficits [87] have been linked to prefrontal NMDA hypoactivity; finally, an excess of glutamate in the hippocampus was associated to dysfunctional executive function (measure with the Wisconsin Card Sorting Test -WCST).

### 4 Glutamatergic and Connectivity WM Hypotheses Are Intrinsically Associated

The main studies tying WM and glutamate are relative to periventricular leukomalacia (PVL), which is an injury to WM that affects the developing oligodendrocites of some newborns and leads to abnormal myelination. This condition is highly associated with hypoxic-ischemic brain injuries associated with premature deliver (before 32 weeks of gestation), which leads to a high concentration of glutamate within the immature brain [42]. A recent study showed that memantine, a NMDAR non-competitive antagonist, attenuates the death of oligodendrocites in WM [88].

In the field of schizophrenia research, it is well established that proneness to schizophrenia can be due to multiple risk factors, genetic, epigenic and developmental, either isolated or in association. Thus, genetic risks can interact with contingent risk factors, like obstetric complications, among which the main variable is represented by hypoxic events [89-91]. In that sense, there might be a significant relation between developmental risks for schizophrenia and for PVL (although studies are needed to confirm this), suggestively relating to structural abnormalities among WM precursors oligodendrocites and affected by excessive glutamatergic exocytose due to obstetric injuries. This is in line with studies that suggest that glial asthenia predisposes to schizophrenia [92, 93], and, even further, with a genetic study that found an association between genes regulated by hypoxia and vascular brain functions, obstetric complications, and the onset of schizophrenia [94].

Moreover, it is worth considering the following picture: over the last few decades, many genes were assumed as possible risk factors for the disorder and then disconfirmed by independent studies. This might not be misleading, but rather express the fact that each of these genes in question increase the chances of schizophrenia only by a little, and make the subject more suitable to the effect of other risk factors (e.g. hormonal imbalance, chronic stress, use of drugs), which are not necessarily present in every case. In the same sense, rare structural genetic variants, represented by novel deletions and duplications of genes are increased (by three fold) among the individuals suffering with schizophrenia [95], and sporadic schizophrenia might be associated with rare *de novo* copy number (CN) mutations [96].

This means that it is not correct to assume that specific genes lead to schizophrenia. Nevertheless, one thing that we actually do know is that some genetic risk factors are more strongly related to the syndrome than most of the others.

One of these important genes is Neuregulin-1 [97-104], which is located in the chromosome 8p13 and drives the production of one of the four proteins of the Neuregulin family (abbreviated by NGR-1). It might be the most important one that is related to the endophenotype of the disorder [105]. With a focus on the molecular interactions that involve Neuregulin-1 (and its ERB receptors), it is possible to demonstrate a strong association between the glutamatergic hypothesis and WM abnormalities, which remain remarkably unexplored in the literature on the matter.

A well known post-mortem study [106], associated NRG-1-ERB signaling to NMDA hypofunction (they highlighted the possible role of ERB-4); notably, the levels of NRG-1 and ERB4 were normal. In the same sense, the team from deCODE company, which had previously suggested Neuregulin-1 as a candidate gene for the syndrome, proposed that Neuregulin-1 would participate in the phosphorylation of one of the subunits (NR2B) of the NMDAR receptors [107]. Also supporting the assumption that Neuregulin-1 is chiefly associated with the glutamatergic hypothesis, a famous study conducted by Stefansson and collaborators [102] concluded that, among rats, the NR-1 hypomorphs have fewer functional NMDA receptors than controls.

On the WM side, one study with rats suggested that NRG-1 is necessary to the survival of oligodendrocytes [108], while another [109] (also with rats) confirmed that Neuregulin-1 is associated with oligodendrocites integrity, and extended this perspective to the associated WM thickness and thus the speed of information transmission (also by the signalization of the NRG-1 receptor erbB4). The authors also suggested that this would cause increased levels of dopaminergic function. That is, they indirectly assumed an hierarchy of neurophysiological abnormalities based on genetic findings, whereas the glutamate cascades should be posited in a higher level of importance, in relation to the dopaminergic cascades.

Even more remarkable with the association that we are proposing was the recent discovery that the haplotype SNP8NRG221533 of the Neuregulin-1 gene carries out its effects over the medial frontal WM in humans [97]. As the authors emphasize: “Our findings add additional support to the notion that NRG1 contributes to myelination and neurodevelopment and that genetic variations in the gene may contribute to disturbed myelination during neurodevelopment in schizophrenia” ([97], p. 716). 

In relation to the studies attempting to define endophenotypic markers of the syndrome, a striking finding was that the Neuregulin-1 gene relates to the P300 wave latency [110], associated with the velocity of neural transmission. This finding led the authors to suggest that the P300 effect could be due to a disruption in WM’s integrity.

In essence, the Neuregulin-1 gene and the molecular cascade NRG-1-ERB are assuming a central role among the variables that participate in the consolidation of schizophrenia’s endophenotype, while being independently related to the glutamatergic and the connectivity WM hypothesis. In terms of the latter, it is prominent that, besides the fact that excessive glutamate exocytose is toxic to the axonal tracts, Neurogulin-1 participates in myelination and, probably, in the firing of WM action potentials.

### 5 Epistemological Basis of a Unified Neurobiological Model of Schizophrenia: Two Levels of the Information Processing Paradigm 

The aforementioned findings suggest that the same risk factors underlie events that occur within the synapses and the transmission of the action potentials across (and within) the WM, which integrates not only nearby cells, but separated networks. In this sense, the integration of the connectivity WM approach with the glutamatergic hypothesis is natural. Moreover, it becomes specifically relevant, as we consider that oligodendrocites-related genes like the 2',3'-cyclic nucleotide 3'-phosphodiesterase (CNP) and the oligodendrocyte-lineage transcription factor 2 (OLIG2) were found to have a similar expressions within schizophrenic patients and controls [111], and thus might not be directly implicated in the syndrome’s etiology.

Nevertheless, this association is insufficient to advance a strong correlation between neurophysiology and neuroanatomy, as we had proposed ourselves to deliver. To achieve this, we need to define the aforementioned ‘dialectic relation’.

Although it is not canonical in the field of theoretical psychopathology, the integration of neuroanatomy and neurochemistry is self-evident; just to mention an example, we can notice that long term exposition to some neurotransmitters and other natural compounds can produce different levels of damage to the cells and even cell death (either by intoxication or by apoptosis). The effects are progressive and can lead to common anatomical abnormalities found in the syndrome (ventricle enlargement, loss of global grey matter volume, etc.). In the opposite direction, a decreased number of specific cells, determined by any other reason can generate a neurochemical imbalance. This explains the fact that, although most of the neurons that are lost are not replaced, drugs can have some stabilizing effect. Thus, one could think: why is this approach not common? Why aren’t the anatomical (represented by cell death) and the neurochemical approaches unified to better characterize the leitmotiv of schizophrenia’s onset and progression? The answer is simple: because depending on the paradigm that we are using, there is no theoretical gain in that perspective.

To define the essence of the epistemological problem that we are facing and to extract a theoretical advantage, we should have in mind the fact that schizophrenia is a disorder that in the first instance affects more acutely localist specific networks. That is, neuronal assemblies that are close related, whereas ‘small-worldness’ becomes moderately impaired (in association with at-risk and onset abnormalities) by means of neurotransmission dysfunction. In a second moment, more pronounced WM abnormalities emerge, leading to an acute decrease in transmission power among neurons from different motifs.

### 6 The Integration of the Glutamatergic Hypothesis with the Disconnection Hypothesis

The brain exhibits a characteristic known as cognitive reserve, which is sometimes defined as an overload of CNS neurons in relation to those that would be indispensable in youth, and sometimes represented by neuropsychological measures of higher cognitive achievement in youth, which are associated with a better prognosis in case of senile brain disorders. Among the elderly, cognitive reserve was demonstrated to be protective of Alzheimer and Dementia [112-115].

In our field of interest, it has been shown that cognitive reserve is smaller in schizophrenia, both as a neuropsychological measure [116] and a physiological measure (brain and intracranial sizes; no reduction was found in extracranial size) [117]. This suggests that the loss of neurons could have a particularly acute effect over those subjects at risk of developing the syndrome. Moreover, this perspective probably relates to the endophenotypes of the on-risk group, and to the fact that some types of cognitive deficits that accompany the syndrome precede its onset.

Remarkably, the extent of grey matter loss among first episode non-medicated patients correlates with measures of glutamatergic metabolites in specific sites of the brain [118]. This finding correlates with the perspective that glutamine/glutamine levels were increased in the medial temporal areas of a cohort of adolescents at a high risk of developing the syndrome [119].

As the above picture suggests, glutamate and associated neurotransmitter cascades become impaired before the onset of the syndrome, and that associated variables (e.g. high levels of stress, hormonal changes) accompany the onset and the structural abnormality (a death) of a substantial number of neurons in local sites of the brain, what turns out to be specially severe considering that the cognitive reserve is reduced.

This situation moderately diminishes small-world indexes in the firstly affected neuronal populations, as we can grasp from a study that showed that increased neuron-neuron connectivity (among rat hippocampus cells) is determined by NDMAR activity, and that NMDAR blockers (ketamine) diminished local connectivity [120]. Thus, it is suggested that this condition over emphasizes the importance (functionality) of network integration, much in the same sense that someone who cannot solve a problem using one strategy, intuitively grasps another. This integrative capacity is only possible due to the availability of functional connectors. “(…) Abnormal experience-dependent plasticity, leading to changes in synaptic density or dendritic arborization, could be reflected by abnormal inter-regional covariation of gray matter volume, even in the absence of macroscopic abnormalities of WM tracts (…)” ([121], p. 9246).

Furthermore, as Price revealed, connectivity (measured over the corpus callosum) is normal at the onset of the syndrome [122]. A recent study that used, as regions of interest, not only the corpus callosum, but several other pathways, also reached the conclusion that WM is not severely impaired at the moment of onset, but becomes impaired in association with chronicity [123]. While another recent study [124] has shown that WM’s fractional anisotropy within fronto-temporal tracts decreases with age after the onset of schizophrenia, while remaining stable among controls. 

But, as we have seen, this neurochemical imbalance that affects grey matter starts to tackle the structure of the WM, not only due to a toxic over exposure to glutamate, but in face of the fact that NRG-1 cascades become impaired all over the brain. 

This ‘circulatory problem’ entraps the individual’s fitness, as it produces a neuropsychological situation in which the brain’s motifs, which were previously and selectively impaired, also become gradually disintegrated. That is the most striking counterpart of the finding that the severity of the topological abnormalities within small-worldness correlate with the duration of the syndrome [125].

By that means, there is a subtle decrease in the functioning level (cognitive, social and subjective), while the experience of the self also becomes acutely unstructured, leading to feelings of depersonalizations and other signs of personality disintegration. Thus, what could be a transient decompensation turns out to be the first-episode of a chronic disorder, whereby cognitive, positive symptoms and negative symptoms express the fact that small-world indexes are reduced to some extent within specific motifs, and the plasticity to use and integrate different motifs is increasingly obstructed. In the end, it is this last aspect that underpins chronicity and determines its severity, as it equates different levels of network impairments in a net fragmentation, which is beyond any level of reduced small-worldness and well-defined cognitive impairments.

## CONCLUSION

Information processing in the human brain can be divided into primary information processes, defined by the rate of firing of action potentials and information transmission, defined by the relation between each two cells and from brain motifs to brain motifs. Neurotransmitters/neuroreceptors can be considered as part of the information transmission domain, much as WM, while it is also true that excessive exocytose of neurotransmitters can be toxic and lead to cell death, and thus, to abnormal primary information processing.

Schizophrenia is a syndrome characterized by the fact that neurotransmitter abnormalities forego noticeable WM abnormalities. The former, chiefly represented by NMDAR hypofunction and associated molecular cascades, are related to the first signs of structural neuronal abnormalities and cell death, within specific brain motifs. The reason for this motif/neuroreceptor association is not totally understood, while the net cost of this situation is a decrease in small-world indexes in related brain areas.

This process is both directly and indirectly integrated to the underpinning of WM structural abnormalities; not only is the excess glutamate toxic to WM, but its disruption is associated with at least one common genetic risk: Neurogulin-1. That represents the first level relation between WM and glutamatergic hypotheses.

Considering the existence of glutamatergic chemical synapses in WM and other aforementioned findings, we can hypothesize that WM abnormalities might be triggered by NMDA hypofunction, which introduces a state of excessive levels of free glutamate - which is toxic to the cells - therefore creating the basis for acute WM abnormalities.

Across the whole brain, WM connectivity is displayed in different manners: as localist indexes of small-world relation within specific brain motifs; connecting directly some of those motifs; and connecting integrative neuronal assemblies. The diminishing small-world indexes within localist networks assigns the importance of overlying different motifs and integrative assemblies, as this is represented to the affected individual, the possibility to overcome the dangers of a drastic disability, maintaining himself/herself to be attached to paradigms of a normal life.

As WM integrity also becomes significantly impaired, this possibility is denied. As a result, the affected individual starts to face a situation of increasing chronicity, whereas his/her condition evolves from specific cognitive deficits associated with psychological reactivity, to general disintegration of cognitive abilities and personality.

## Footnotes

1 More recently interest has also been driven to the metabotropic G-protein coupled receptors (GLU).
